# Prevalence and Risk Factors of Human Papillomavirus (HPV) Infection in Southern Chinese Women – A Population-Based Study

**DOI:** 10.1371/journal.pone.0019244

**Published:** 2011-05-03

**Authors:** Stephanie Si Liu, Kelvin Yuen Kwong Chan, Rebecca Ching Yu Leung, Karen Kar Loen Chan, Kar Fai Tam, May Hiu Mei Luk, Sue Seen Tsing Lo, Daniel Yee Tak Fong, Annie Nga Yin Cheung, Zhong Qiu Lin, Hextan Yuen Sheung Ngan

**Affiliations:** 1 Department of Obstetrics & Gynaecology, The University of Hong Kong, Hong Kong; 2 Department of Pathology, The University of Hong Kong, Hong Kong; 3 School of Nursing, The University of Hong Kong, Hong Kong; 4 Family Planning Association of Hong Kong, Hong Kong; 5 Department of Obstetrics & Gynaecology, The Second Affiliated Hospital of Sun Yat-Sen University, Guangzhou, China; Karolinska Institutet, Sweden

## Abstract

**Background:**

Persistent high-risk type Human papillomavirus (HPV) infection is recognized as a necessary cause of cervical cancer. This study aimed to compare the HPV prevalence and risk factors between women residing in Hong Kong (HK) and Guangzhou (GZ) region of China.

**Methodology/Principal Findings:**

A total of 1,570 and 1,369 women were recruited from HK and GZ, respectively. The cytology samples were collected and tested for HPV infection. The overall and type-specific HPV prevalence and the potential risk factors for acquisition of HPV infection were studied. Women with normal cytology in the GZ cohort had significantly higher HPV prevalence (10%) than those in the HK cohort (6.2%, p<0.001). The patterns of the age-specific HPV prevalence were also different between the two cohorts. In the HK cohort, women at the age of 20–29 years old had the highest prevalence and a second peak was observed in the age of ≥60 years old. In the GZ cohort, the highest HPV prevalence was also observed in 20–29 years old but declined as the age increased and a second peak was not seen. HPV16 and HPV52 were the most common high-risk types found in the HK and GZ cohorts, respectively. Age was the most consistently observed independent risk factor for HPV infection in the HK, while the number of sexual partners had association in the GZ cohort.

**Conclusions/Significance:**

Our study provides the current status and the epidemiological characteristics of HPV prevalence in Southern Chinese women. The results strongly suggested that population education and the effective cervical cancer screening would be vital in the prevention of cervical cancer.

## Introduction

Cervical cancer is the second most common woman cancer worldwide, but it is ranked first in South Asia [1]. Epidemiological and molecular studies have shown that genital HPV is the main etiological factor for the disease [2], [3]. HPV is a common sexually transmitted pathogen that plays an important role in the pathogenesis of pre-cancerous cervical lesion and cervical cancer [2], [4]. More than 100 types of HPVs have been identified and about 40 of them are found associated with lesions of the female genital tract. Persistent infection with one of 13 high-risk HPV types causes almost all cervical cancers [5]. Knowledge of HPV status becomes more important as it can be used as a primary screening method for cervical cancer, and for triage of women with atypical or borderline cervical smears.

Analysis of worldwide distribution of HPV types on the four continents, Asia, Africa, Europe and South America, showed that HPV prevalence varied as much as 20 times among populations in different geographic regions [5], [6]. Focusing on the Southeast Asian countries, the prevalence ranged from 1.6% in Hanoi Vietnam to 13.3% in Korea [6]. Within the Chinese populations, HPV prevalence and subtype distribution in cervical lesions and invasive cancer differed amongst different geographic areas in mainland China [7]–[9]. The prevalence of certain HPV types in Hong Kong (HK), especially HPV16 variants, was also different from that reported in Sichuan, China [10]. The HPV16 isolated from Sichuan had the closest similarity to Asian-American and Asian lineages [11], whereas the most prevalent HPV16 variant found in HK were of Asian and European lineages [12]. HK has been a British colony and it had been the gateway for trading with the West for centuries. This may explain the prevalence of European variants in this area.

The incidence and mortality of cervical cancer were ranked the fifth and the ninth respectively amongst all cancers in HK women in 2006. There were 459 new cases and 133 death reported. Although a decline in incidence and mortality of cervical cancer was reported in mainland China in the 1970s [13]–[16], the mortality was found increased in young women, particular in urban areas, which was suggested to be a reflection of the changes in the sexual behaviours [16]. This was evident by the increased incidence of sexually transmitted diseases in China in 1990s [17]. The epidemiology of HPV genotypes in Guangzhou (GZ), one of HK's closest neighbouring cities, has not yet been studied. Following the economical growth and improved transportation between HK and mainland China, especially the GZ regions, cross-boundary marriages and travelling have tremendously increased in the last decade. As a result, common infectious diseases are easily transmitted across the border. In the present study, we aimed to study and compare the spectrum and the prevalence of HPV infection in women living in HK and GZ regions, and to identify potential risk factors in both regions.

## Results

### Characteristics of the study population

A total of 1570 and 1369 women were recruited in HK and GZ, respectively. Those with abnormal cytological findings (44 in HK and 69 in GZ) were excluded from the study. HPV prevalence analyses were performed on 1526 (HK) and 1330 (GZ) cases, whose genomic DNA was successfully extracted for HPV detection and genotyping, and the correlation analyses were performed on 1330 (HK) and 1233 (GZ) cases as these cases had all of the potential risk factors data available for statistical analysis.

Most women in both HK and GZ cohorts had intermediate (secondary/post-secondary) or high (college/university or above) education levels (78% in HK and 89% in GZ), had their first sexual activity before the age of 25 (76% in HK and 67% in GZ) and one lifetime sexual partner (69% in HK and 86% in GZ). Few women smoked (12% in HK and 3% in GZ) or had a history of sexually transmitted disease (<2% in both cohorts). Only 31% of women in the GZ cohort had previous cervical smear test; in contrast, 84% of women in the HK cohort had the test previously. This relative low value in the GZ cohort would be due to the fact that, unlike HK, GZ had no cervical screening programme provided to the community. The use of contraceptive methods was also different between the two cohorts, where 45% and 86% of women used a contraceptive method other than the oral contraceptive pills. Two thirds of women in the GZ cohort had a previous termination of pregnancy or miscarriage (69%), while one third was observed in the HK cohort (34%).

### HPV prevalence in the Hong Kong and Guangzhou cohorts

**Figure 1:**
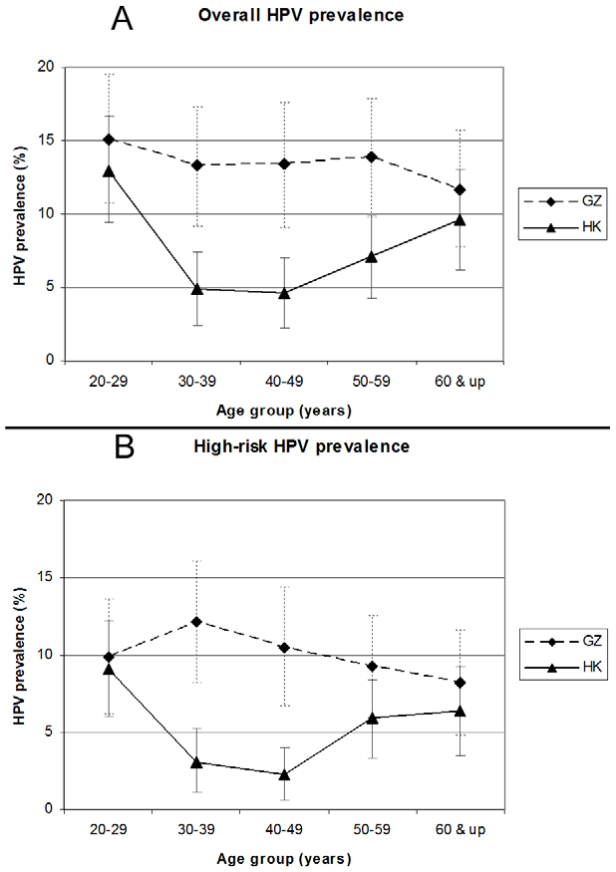
Comparison of the age-specific overall and high-risk HPV prevalence in five age-groups in the HK and GZ cohorts. (A) Overall HPV prevalence: significant difference in the overall HPV prevalence between the two cohorts in age-groups of 30–39, 40–49 and 50–59 (p<0.001, p<0.001 and p = 0.007, respectively, z test). (B) High-risk HPV prevalence: significant difference in the high-risk HPV prevalence between the two cohorts in age-groups of 30–39 and 40–49 (p<0.001 and p<0.001, respectively, z test). The error bar indicates 95% confidence interval.

The overall HPV prevalence in women with normal cytology between the two cohorts was significantly different (6.2%, 95% CI: 5.1–7.3% for HK and 10%, 95% CI: 8.4–11.5% for GZ, p<0.001). The middle-aged groups (30–59 years old) in the GZ cohort had significantly higher HPV prevalence (p<0.001-p = 0.007) than that in the HK cohort (Fig. 1A). In the GZ cohort, the 20–29 age-group was found to have the highest HPV prevalence (15.1%, 95% CI: 10.7–19.5), and the prevalence declined as the age increased, and there was no significant difference observed in HPV prevalence among the five age-groups. Interestingly, a two peak (bimodal distribution) pattern of HPV prevalence was observed in the HK cohort. Women below the age of 29 had the highest HPV prevalence (13%), which was significantly higher than that in the middle-aged women among age-groups of 30–59 years old (HPV prevalence of 4.9% for 30–39, 4.6% for 40–49, and 7.1% for 50–59 age-groups, p = 0.0003, 0.0001, and 0.011, respectively), and there was a less pronounced second peak of HPV infection (Fig. 1A) in women over the age of 60 years (9.6%), which was significantly higher compared to women in the 30–39 age-group (4.9%) (p = 0.029).

High-risk HPV types comprised more than 2/3 of the HPV infection in both of our cohorts. The GZ cohort had significantly higher high-risk type HPV prevalence (7.5%) than those in the HK cohort (4.2%, p<0.001). The patterns of high-risk HPV prevalence were similar to that of overall HPV prevalence in the two cohorts (Fig. 1B), a significant difference between the two cohorts was observed in women in the 30–49 age-groups (p<0.001). In the HK cohort, the high-risk type HPV prevalence also followed a bimodal distribution with the highest peak appearing in the 20–29 age-group (9.1%) and the second peak appearing in ≥50 age-groups (5.9%–6.4%). The 30–39 and 40–49 age-groups had significantly lower prevalence (3.1% and 2.3%, respectively) than other age-groups (p = 0.02–0.002). No significant difference in the high-risk HPV prevalence was found among the five age-groups in the GZ cohort, and the highest prevalence was in 30–39 age-group.

The prevalence of multiple HPV infections was 1.8% and 1.7% for the HK and GZ cohorts, respectively, accounting for 30.6% and 16% of the overall HPV positive cases. The highest prevalence of multiple HPV infections was also found in 20–29 age-group (4.2%) and ≥60 age-group (3.6%) in the HK cohort, whereas the prevalence was appeared to be the highest in the 20–29 age-group (3.2%) in the GZ cohort, while it declined in other age-groups (2.7%, 1.2%, 2.5% and 1.2% in age-groups of 30–39, 40–49, 50–59, ≥60, respectively).

### HPV type-specific prevalence

The HPV type-specific prevalence was also different between the two cohorts. While HPV16 being the most common high-risk HPV type (1.24%, 95% CI: 0.75–1.73%, Table 1) identified in the HK cohort, HPV52 was the most prevalent in the GZ cohort (2.62%, 95% CI: 1.75–3.48%). The two HPV types contributed to be the most common high-risk types in the two cohorts. The seven most common high-risk HPV types observed in the HK cohort were HPV16, 52, 58, 18, 33, 68 and 45 (in descending order) and in the GZ cohort were HPV52, 16, 31, 58, 18, 33 and 68 (in descending order), respectively (Table 1). HPV31 was not detected in HK cohort and the HPV52 prevalence in the GZ cohort was significantly higher than that in the HK cohort (p = 0.004).

**Table 1:** The prevalence of type-specific HPV infection in women with normal cytology in the HK and GZ cohorts.

	Hong Kong		Guangzhou		
HPV type	No. of positive case	Prevalence** (95%CI)	No. of positive case	Prevalence** (95%CI)	P value*
16† ‡	25	1.24% (0.75–1.73)	21	1.19% (0.63–1.76)	0.901
18† ‡	20	0.99% (0.56–1.43)	9	0.68% (0.22–1.15)	0.339
31‡	0	0	27	1.16% (0.67–1.66)	**<0.001** *
33† ‡	5	0.25% (0.03–0.47)	10	0.59% (0.2–0.98)	0.136
35	0	0	1	0.07% (0.0–0.21)	0.317
39	1	0.04% (0.0–0.12)	6	0.32% (0.03–0.6)	0.07
45†	3	0.15% (0.0–0.31)	3	0.24% (0.0–0.54)	0.577
51	0	0	2	0.14% (0.0–0.35)	0.198
52† ‡	23	1.16% (0.68–1.64)	40	2.62% (1.75–3.48)	**0.004** *
56	0	0	4	0.27% (0.0–0.54)	0.058
58† ‡	20	1.03% (0.58–1.48)	18	1.07% (0.53–1.62)	0.905
59	2	0.09% (0.0–0.21)	2	0.15% (0.0–0.37)	0.619
68† ‡	5	0.23% (0.03–0.44)	7	0.39% (0.06–0.72)	0.428

*Statistically significant at 0.05 level of significance by Holm's procedure that accounts for the multiple comparisons.

**Adjusted for the stratified sampling design.

† The seven commonest HPV high-risk types in the HK cohort.

‡ The seven commonest HPV high-risk types in the GZ cohort.

### Risk factors for HPV infection

Potential risk factors in relation to overall HPV infection were also examined. Age was found to be a significant risk factor associated with HPV infection in the HK cohort (p = 0.005, Table 2). Women in the 20–29 age-group had significantly higher risk for HPV infection compared to 30–39 and 40–49 age-groups (OR = 0.39, 95% CI: 0.19–0.8 and  = 0.42, 95% CI: 0.18–0.98, respectively). On the other hand, only 16% of women in the HK cohort had no previous cervical smear test and these women had a trend of having higher risk for HPV infection (OR = 1.82 95%CI: 0.99–3.34) than those having history of the smear test performed; however, the association did not reach statistical significant level. Similarly, despite not reaching the statistical significant level, women lacking previous cervical smear test in the GZ cohort tended to have higher risk for HPV infection (OR = 1.43, 95% CI: 0.97–2.11).

**Table 2:** Potential risk factors in relation to HPV infection in the HK and GZ cohorts.

	Hong Kong^1^ (N = 1330)				Guangzhou^2^ (N = 1233)			
Variables	No. of case (%)	No. of HPV+	OR^3^ (95% CI)	P value^3^	No. of case (%)	No. of HPV+	OR^3^ (95% CI)	P value^3^
**Age (years)**				**0.005**				0.688
20–29	298 (22.4)	37	1		236 (19.1)	36	1	
30–39	260 (19.5)	13	0.39 (0.19–0.8)		240 (19.5)	34	1.25 (0.68–2.27)	
40–49	252 (18.9)	12	0.42 (0.18–0.98)		231 (18.7)	27	1.06 (0.55–2.05)	
50–59	262 (19.7)	16	0.67 (0.27–1.7)		271 (22)	38	1.45 (0.74–2.83)	
⇒60	258 (19.4)	25	1.3 (0.5–3.4)		255 (20.7)	30	1.06 (0.52–2.14)	
**Screening history**				0.054				0.075
Pap smear	1114 (83.8)	79	1		384 (31.1)	41	1	
No Pap smear	216 (16.2)	24	1.82 (0.99–3.34)		849 (68.9)	124	1.43 (0.97–2.11)	
**Lifetime number of sexual partners**				0.877				**0.041**
1	919 (69.1)	63	1		1063 (86.2)	131	1	
2	202 (15.2)	18	1.12 (0.59–2.13)		124 (10.1)	20	1.42 (0.81–2.48)	
⇒3	209 (15.7)	22	1.19 (0.59–2.36)		46 (3.7)	14	2.61 (1.2–5.68)	
**Age at first intercourse**				0.351				0.842
⇐20	469 (35.3)	50	1		177 (14.4)	27	1	
21–25	536 (40.3)	31	0.67 (0.39–1.18)		654 (53)	90	1.07 (0.64–1.79)	
⇒26	325 (24.4)	22	0.86 (0.44–1.69)		402 (32.6)	48	0.95 (0.51–1.76)	
**Gravidity**				0.115				0.884
0	391 (29.4)	29	1		88 (7.1)	17	1	
1–2	494 (37.1)	44	1.13 (0.55–2.34)		563 (45.7)	70	0.91 (0.4–1.64)	
3–4	344 (25.9)	26	0.69 (0.25–1.85)		459 (37.2)	61	0.92 (0.39–2.17)	
⇒5	101 (7.6)	4	0.28 (0.06–1.22)		123 (10)	17	0.92 (0.3–2.79)	
**Number of abortion**				0.097				0.187
0	884 (66.5)	59	1		388 (31.5)	61	1	
1–2	407 (30.6)	42	1.82 (1.03–3.22)		691 (56)	80	0.67 (0.43–1.05)	
⇒3	39 (2.9)	2	1.1 (0.21–5.9)		154 (12.5)	24	0.8 (0.38–1.69)	
**Condom use**				0.122				0.604
Never or rare	717 (53.9)	64	1		899 (72.9)	123	1	
Regular	613 (46.1)	39	0.7 (0.45–1.1)		334 (27.1)	42	0.9 (0.6–1.35)	
**Oral contraceptives**				0.126				0.556
Never	595 (44.7)	40	1		1055 (85.6)	139	1	
Ever	735 (55.3)	63	1.42 (0.91–2.24)		178 (14.4)	26	1.15 (0.72–1.85)	
**History of STD**				0.259				0.204
Never	1309 (98.4)	99	1		1214 (98.5)	159	1	
Ever	21 (1.6)	3	2.14 (0.57–8.04)		19 (1.5)	6	2.02 (0.68–6.0)	
**Smoking**				0.96				0.98
Never	1175 (88.3)	79	1		1197 (97.1)	158	1	
Former	79 (5.9)	8	0.97 (0.42–2.24)		17 (1.4)	3	1.03 (0.28–3.87)	
Current	76 (5.7)	9	0.89 (0.38–2.05)		19 (1.5)	4	1.13 (0.33–3.9)	
**Education**				0.493				0.55
Nil/primary	289 (21.7)	21	1		135 (10.9)	20	1	
Secondary/post-secondary	717 (53.9)	59	1.13 (0.59–2.15)		597 (48.4)	75	0.9 (0.51–1.61)	
College/University & above	324 (24.4)	23	0.81 (0.35–1.86)		501 (40.6)	70	1.12 (0.6–2.09)	

1 :Tolerance >0.4 for all variables, Naglekerke R^2^ = 7.8%, Hosmer-Lemeshow test p = 0.793.

2 :Tolerance >0.4 for all variables, Naglekerke R^2^ = 3.5%, Hosmer-Lemeshow test p = 0.750.

3 :OR and P-value were obtained using multivariate logistic regression analysis model of which included all of the variables listed in this table. Bold type indicated statistically significant values.

N: Total number of cases.

Sexual behaviour factor, the lifetime number of sexual partners was found to be associated with the risk of HPV infection in the GZ cohort (Table 2). Women with more than two lifetime sexual partners were found to have significant higher risk for HPV infection than women with 1–2 sexual partners (OR = 2.61, 95% CI: 1.20–5.68, p = 0.041). Increase of number of sexual partners tended to have increase of HPV infection risk in the HK cohort (ORs for 2 and ≥3 lifetime sexual partners were 1.12 and 1.19); however, the difference was not statistically significant. Another sexual behaviour factor, age at first sexual intercourse was found not significantly associated with HPV infection risk in both studied cohorts.

The reproductive factors analyzed in the present study seemed to have no influence in HPV infection risk in both cohorts. Ever being pregnant or number of pregnancies, number of abortions, condom and oral contraceptive use had no significant association. Having previous sexually transmitted disease (STD) and being smoker tended to have increase of HPV infection risk, however they did not reach statistical significance. Furthermore, the level of education was not a risk factor for HPV infection in both of the study cohorts.

## Discussion

Our cross-sectional study examined and compared the HPV prevalence in women residing in HK and GZ. The age-adjusted prevalence of HPV infection in women with normal cytology in the HK cohort was significantly lower than that in the GZ cohort (6.2% vs 10%, p<0.001), and this might partly be attributed to the difference in sample population. Samples recruited in HK were from women with regular cervical screening, while samples in GZ were from women without or with only opportunistic cervical screening due to lack of cervical screening programme there. The HPV prevalence in the HK cohort was also lower than the previously reported worldwide data of 10.4% (95% CI 10.2–10.7), but similar to those areas with lower HPV prevalence in the Asia region, such as Thailand, Philippines and India [18]. The age-adjusted HPV prevalence (10%) in the GZ cohort was comparable to the results obtained in other regions of mainland China, such as Shanxi [7], [19], [20]. Besides the prevalence, the age-specific infection patterns were also different between our two studied cohorts. Younger women (20–29 years old) in the GZ cohort had the highest infection and the prevalence was decreased with increasing age, whereas a bimodal distribution of HPV prevalence was observed in age-groups of 20–29 and ≥60 in the HK cohort. Similar to the other part of world, the highest HPV prevalence was seen in the youngest age group in our study, suggesting a higher possibility of acquiring HPV infections at younger ages [21].

Our findings in the HK cohort was also in-line with the reported meta-analysis study showing that HPV infection was most common in young age women (<30 years) and a second peak in women aged 45 or older [18]. The second peak in the HK cohort started late, from 60 years of age. This two-peak pattern is the predominant prevalent curve reported in women with normal cytology worldwide [18]. The phenomenon has been hypothesized to be the results of reactivation of latent infections due to impaired immune response, or changes in sexual behaviors in women and their partners [18]. However, the second peak was not obvious in our GZ cohort. HK and GZ are in closed neighbourhood, the different socioeconomic environment, such as lifestyle and living standards are different between the two regions. This may explain the difference in prevalence pattern. HK used to be a British colony before 90 s. It is a well developed city and the economic and social lives are closely related to those in the Western countries. The prevalence curve in the GZ cohort was also different from those from central and northeastern parts of mainland China, such as Shanxi and Shenyang, where high HPV prevalence was seen across all age groups [7], [22]. The relatively low HPV prevalence compared to other cities in China and the different prevalence pattern observed in GZ may be explained by a relatively low cervical cancer burden in that area [19].

Our current study assessed HPV type-specific prevalence across a broad age-range of the women in Southern China. The type-specific HPV prevalence was also found to be different between the two study cohorts. HPV16 remained to be the most common high-risk type in HK, whereas HPV52 was the most frequently found in the GZ cohort. Our findings were in concordance with the previous reports from some Asian countries where the HPV52 and HPV58 infections were as common as HPV16 infection [23], [24]. Indeed, the infection rate of HPV52 was second to that of HPV16 in the HK cohort, and HPV58 ranked third and fourth in HK and GZ, respectively. There was a significantly higher prevalence of HPV31 in the GZ cohort when compared to that in the HK cohort.

Having previous cervical cancer screening was found to be important in relation to HPV infection in the HK cohort. Although such association was not observed in the GZ cohort, women lacking previous cervical smear was found to be associated with the higher HPV prevalence (Table 2). This suggests that those who do not attend for regular smears test may belong to a group of women with high risk for HPV infection and development of cervical cancer. It is therefore important to identify this group of women and try to target the cervical screening promotion at them.

Various aspects of sexual behaviour were reported to be related to the acquisition of HPV infection. In the present study, only the lifetime number of sexual partners was associated with HPV infection risk in the GZ cohort. Women having three or more lifetime sexual partners had significantly higher risk of HPV infection, suggesting that sexual behaviour, as elsewhere [22], [25], [26], is an important determinant of HPV infection, particularly in the GZ region. The more sexual partners that a woman has, the higher risk of getting infected with one or more HPV types over time. There was no significant association with the number of sexual partners in the HK cohort for HPV infection risk, and the result was different from other local studies, where lifetime number of sexual partners was shown to be an independent risk factor [12], [27]. This may be attributed to the difference in the study population; their study population included women regardless of their cytological findings, whereas only women with normal cytology were included in our study. Since HPV is mainly sexually transmitted, information on the sexual behaviours of the sexual partners may help to explain the difference findings between the two cohorts. Unfortunately, no such information was available at the present study.

Results of the studies regarding the effect of smoking on HPV infection have been inconsistent; some showed that smoking was associated with an increased prevalence of HPV [28], [29], but not others [30]–[33]. We found an increased trend of HPV prevalence in current smokers compared to non-smokers in both cohorts. However, the numbers of current and former smokers at either cohort were not sufficiently large to draw a conclusive finding. Similar situation was also encountered in the association analysis of history of STD in both cohorts.

In conclusion, the overall and type-specific HPV prevalence were different between the HK and GZ cohorts. A difference pattern of the age-specific HPV prevalence was also observed in the two cohorts. Such difference could be due to the difference in life style and socio-economic status between these two neighboring regions. Young age was the most prominent independent risk factor for HPV infection in the HK cohort, while the lifetime number of sexual partner was found to be the infection risk factor in the GZ cohort. Our findings would enhance the understanding of the epidemiology of HPV infection in the two regions, and the information will assist in developing a constructive screening program, the choice of HPV types to be included in the development of the HPV vaccine in future to provide protection against those high-risk types of HPV with high prevalence identified in our cohorts, but not included in the current existing HPV vaccines.

## Materials and Methods

### Ethics statement

The study was approved by the local Institutional Reviewer Board of the University of Hong Kong/Hospital Authority of Hong Kong Western Cluster (HKU/HA HKW IRB, Ref# UW04-267T/589). All participants provided voluntary written informed consent. Information sheets and written consent forms were available in Chinese and English to ensure comprehensive understanding of the study objectives, potential risks, and benefits.

### Study design and sample collection

This was a cross-sectional study between the regions of HK and GZ, China, and was collaboratively conducted amongst Departments of Obstetrics & Gynaecology and Pathology of the University of Hong Kong (HKU), the Family Planning Association of Hong Kong (FPAHK), and Department of Obstetrics & Gynaecology, Second Affiliated Hospital of Sun Yat-Sen University (SYSU), GZ, during the period of January 2007–April 2008. In HK, cytology samples were collected from women attending the clinic in FPAHK for routine cytological screening. Participants were interviewed by a trained interviewer using a standardized questionnaire to elicit information on the demographic variables, cervical screen history, sexual behaviours, contraceptive practice, reproductive history and smoking habits. Informed consent was obtained from each participant. The recruitment was designed to obtain an age-stratified sample groups including at least 250 women in each of the 10-year age group from 20–29, 30–39, 40–49, 50–59, 60 and older. In GZ, similar procedures were carried out and cytology samples were collected from healthy women attending the out-patient clinic in the Department of Obstetrics & Gynaecology, Second Affiliated Hospital of SYSU.

All participants underwent a pelvic examination by a gynaecologist or a trained nurse. The exfoliated cervical cells were collected by ThinPrep™ (in HK) or LCT (in GZ) liquid-based cervical cytology test. The cytological screening tests were performed by the cervical cytology laboratories at the Department of Pathology, HKU and SYSU, respectively.

### HPV detection and genotyping

DNA extraction and HPV genotyping tests were performed in respective institutes where the specimens were collected. DNA was extracted from the cytological remnants, and HPV DNA was detected and genotyped using a commercial GenoArray HPV Genotyping Test (Hybribio, Ltd, Hong Kong) [34].

HPV GenoArray Test is a PCR-based assay and capable of amplifying 21 HPV genotypes including 13 HR types (16, 18, 31, 33, 35, 39, 45, 51, 52, 56, 58, 59, 68). The assay was performed according to manufacturer's protocol. Briefly, after PCR amplification, the amplicons were subjected for hybridization. The assay utilized a “flow-through hybridization” technique by actively directing the targeting molecules toward the immobilized probes within the membrane fibers, and the complementary molecules were retained by forming duplexes. The hybrids were then detected by the addition of streptavidin-horseradish peroxidase conjugate and a substrate (NBT/BCIP). The provided positive control and two negative controls (HPV negative C33-A cells and PCR water) were included in each set of PCR to assess the performance of the test.

### Statistical analysis

HPV types were categorized by oncogenicity: HPV16, 18, 31, 33, 35, 39, 45, 51, 52, 56, 58, 59 and 68 were categorized as high-risk type (HR) and other types were grouped as other risk types. The age-specific general population of HK in 2006 and GZ in 2003 (the latest data available) were used in the calculation of the HPV infection prevalence and the age distribution of women with normal cytology was assumed to be similar to the general population. The prevalence of overall HPV infections, type-specific HPV infections and multiple infections as well as their corresponding 95% confidence intervals (CIs) among HK and GZ women with normal cytology were estimated after taking account of the stratified random sampling design [35]. The HPV prevalence between the HK and GZ cohorts were compared by a z-test. Multicity was accounted by using the Holm's procedure [36]. The effects of epidemiologic variables on HPV infection in women with normal cytology were assessed by univariable and multivariable logistic regression with age-groups of 10 years interval (20–29, 30–39, 40–49, 50–59, 60 and up) independently in the HK and GZ cohorts, and the odd ratios and their 95% CIs were calculated. Multi-collinearity in the multivariable analysis was assessed by examining the tolerance and the goodness of fit of the logistic models were assessed by the Hosmer-Lemeshow test. A P-value<0.05 was regarded as statistically significant. The statistical analysis was performed using SPSS version 16.0.
